# Accurate Prediction of DnaK-Peptide Binding via Homology Modelling and Experimental Data

**DOI:** 10.1371/journal.pcbi.1000475

**Published:** 2009-08-21

**Authors:** Joost Van Durme, Sebastian Maurer-Stroh, Rodrigo Gallardo, Hannah Wilkinson, Frederic Rousseau, Joost Schymkowitz

**Affiliations:** VIB Switch Laboratory, Vrije Universiteit Brussel, Brussels, Belgium; University of Rome, Italy

## Abstract

Molecular chaperones are essential elements of the protein quality control machinery that governs translocation and folding of nascent polypeptides, refolding and degradation of misfolded proteins, and activation of a wide range of client proteins. The prokaryotic heat-shock protein DnaK is the *E. coli* representative of the ubiquitous Hsp70 family, which specializes in the binding of exposed hydrophobic regions in unfolded polypeptides. Accurate prediction of DnaK binding sites in *E. coli* proteins is an essential prerequisite to understand the precise function of this chaperone and the properties of its substrate proteins. In order to map DnaK binding sites in protein sequences, we have developed an algorithm that combines sequence information from peptide binding experiments and structural parameters from homology modelling. We show that this combination significantly outperforms either single approach. The final predictor had a Matthews correlation coefficient (MCC) of 0.819 when assessed over the 144 tested peptide sequences to detect true positives and true negatives. To test the robustness of the learning set, we have conducted a simulated cross-validation, where we omit sequences from the learning sets and calculate the rate of repredicting them. This resulted in a surprisingly good MCC of 0.703. The algorithm was also able to perform equally well on a blind test set of binders and non-binders, of which there was no prior knowledge in the learning sets. The algorithm is freely available at http://limbo.vib.be.

## Author Summary

Molecular chaperones are essential elements of the protein quality control machinery that governs translocation and folding of nascent polypeptides, refolding and degradation of misfolded proteins, and activation of a wide range of client proteins. This variety of functions results from the existence of multiple chaperones with different structures. Chaperones bind to exposed regions of proteins to fulfil their function. The chaperone must hereby recognise a certain signal sequence on the substrate protein. The nature of the sequence that is exposed will determine the types of chaperones that can interact with it, and in the end will also determine the fate of the substrate protein: refolding, translocation, degradation or activation. Knowledge of the binding sequence determinants of molecular chaperones will shed more light on the mechanism of how each chaperone contributes to the cellular protein quality control system.

In this study we have made an algorithm which accurately predicts binding sites for the well studied *E. coli* Hsp70 chaperone, DnaK, which is implicated in folding efficiency and prevention of aggregation. The ability to detect and design high-affinity DnaK binding sites enhances our understanding of chaperone-substrate recognition and opens great opportunities to enhance protein solubility using protein-DnaK binding motif fusions.

## Introduction

Hsp70 molecular chaperones are part of the quality control machinery that functions to assist protein folding. Members of the Hsp70 family have been implicated in refolding of misfolded proteins, folding of newly synthesized polypeptide chains, disassembly of larger aggregates and translocation of proteins in organelles [1]. Hsp70 molecules also enable cell survival during stress or heat-shock conditions that are characterized by an increased concentration of (partially) denatured polypeptides. These chaperones recognize and bind misfolded or aggregation-prone peptide stretches through exposed hydrophobic regions which are normally buried in the protein core. Such exposed regions are typical for non-native proteins [2],[3].

Hsp70 molecular chaperones consist of two distinct domains, an N-terminal ATPase domain [4] and a C-terminal peptide binding domain [5]. Hsp70 function is dependent on an ATP-regulated cycle of substrate binding and release [6]. With ATP bound, substrate affinity is low and Hsp70 resides in an open state, ready to receive a suitable substrate. Once the substrate is bound, ATP is hydrolyzed to ADP and Hsp70 undergoes a conformational change to a high affinity state, subsequently trapping the substrate. The co-chaperone Hsp40 (DnaJ in *E. coli*) binds Hsp70 and stimulates the ATPase function, causing retention of the substrate. Hsp40 also recognizes hydrophobic stretches and may serve as a substrate delivery chaperone to Hsp70 [6],[7]. Upon exchange of ADP for ATP, Hsp70 returns to a low affinity state, enabling binding of another substrate or providing another refolding cycle for the same substrate if necessary.

The crystallisation of the archetypical and well characterized *E coli* Hsp70 DnaK bound to a peptide reflects a heptameric substrate binding motif requiring a hydrophobic core region and preferably basic flanking residues that complement the overall negatively charged DnaK surface [5]. This preference was later confirmed in the seminal work of Bukau and co-workers by binding studies of DnaK to cellulose-based peptide libraries and a DnaK binding profile was derived [3].

Contrary to these previous studies on DnaK binding motif profiling which utilised only sequence information, we complement the experimental binding information on a set of peptides with structural data from homology modelling to obtain an accurate predictor. Similar dual based approaches have already been shown successful to predict other peptide signatures. Prediction of binding of endogenous antigenic peptides to MHC class I molecules was aided by adding structural information from molecular models to the sequence data [8]. Branetti *et al* used structural data from various SH3/ligand complexes and sequence information from phage libraries to predict preferred ligand binding to different SH3 domains [9]. Recently, an algorithm to predict amylogenic regions in protein sequences profited greatly from the combination of sequence based data and structural information from amyloid fibers crystallographic studies (Maurer-Stroh *et al*, unpublished data).

In this article we introduce such a dual based method for profiling DnaK binding sequences. We combine sequence based information from experimental binding assays with structural information from molecular modelling via the FoldX force field [10]. We present a DnaK binding prediction algorithm that, under cross-validated conditions, performs strikingly accurate.

## Results

### Peptide set composition

**Figure 1:**
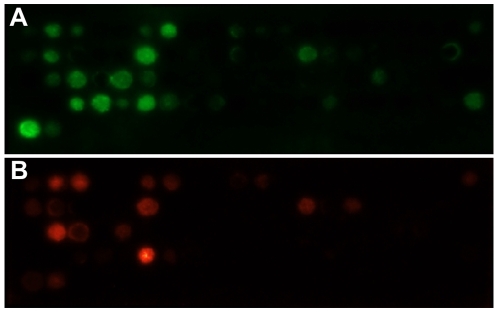
DnaK binding to immobilised peptides attached to a cellulose-based membrane. A) Group 2 peptides Membrane A, B) Group 2 peptides Membrane A bound to antibody only, indicating potential false positives (see supplementary Table 1 for all groups of peptide sequences and raw binding data).

We screened the ability of DnaK to bind cellulose-immobilised peptides by detecting the bound DnaK *via* a specific monoclonal antibody (see materials and methods), according the method previously developed by Bukau and co-workers [3]. Figure 1 shows the outcome of a typical experiment. The peptides were selected in groups by different criteria. Group 1 peptides were selected using the statistical thermodynamics algorithm TANGO for the prediction of cross-beta protein aggregation [11], on the assumption that the Hsp70 chaperone family binds to exposed sequences that can nucleate protein aggregation. An initial crude DnaK predictor was then constructed on the basis of these peptides. The *E coli* proteome was then scanned with this predictor to search for relatively short proteins harbouring more than one predicted DnaK binding sites. Seven proteins that met these requirements were randomly picked for a DnaK binding peptide scan and were named Group 2. Such a peptide scan consists of subsequent decapeptides overlapping by five residues, spanning the whole protein sequence. Two additional putative DnaK binding peptides from the known DnaK binding RNA-polymerase sigma-32 factor [3],[12] were included in the binding experiments (Group 3). All analyzed peptide sequences are listed in Table S1 of the Supplemental data.

A separate experiment was carried out where the peptide membrane was drenched with only the monoclonal antibody but not DnaK. A significant chemiluminescence signal would be the result of antibody-peptide binding and thus a false positive. A total of 16 peptides with a false positive signal higher than half of the DnaK-peptide signal were removed from the sets for any further analysis. The false positive signal of the rest of the peptides was subtracted from their DnaK-interaction signal to correct for any antibody-peptide binding that might occur.

To build the learning sets of sequences (see below), the peptides were grouped as either binders or non-binders. Setting a single cut-off value of relative binding would create a twilight zone of peptides that show neither clear binding nor clear non-binding. Therefore we set a high and a low cut-off value per membrane/experiment. Peptides with a signal above the high cut-off are considered as binders, peptides with a signal below the low cut-off are considered as non-binders.

Out of a total of 172 peptides, there were 53 binders and 119 non-binders. A fraction of 15% from each set was randomly selected to constitute an independent validation set. Sequences from this validation set were never implemented in any training sets of the predictor to allow an independent validation assessment. This set consisted of 9 binders and 19 non-binders. The remaining 144 peptide sequences will be referred to as ‘benchmark’ sets. The binders and non-binders groups are less then 90% redundant in sequence identity.

### Learning set selection

A sequence based profile or position specific scoring matrix (PSSM) can only be derived from properly aligned learning sets of sequences of the same length. As the aim of our DnaK predictor was to evaluate stretches of heptapeptides for DnaK binding affinity, we fragmented both positive (binders) and negative (non-binders) benchmark sets into heptapeptides to create the final learning sets. To generate the negative learning set we assumed that for peptides longer than 7 residues, each heptapeptide fragment of the peptide is a true non-binder. Therefore the final negative learning set consisted of all possible heptapeptides from the full length non-binding peptides.

Generating a heptapeptide learning set from the true binder peptides is less trivial as one or more heptapeptide stretches must be identified from each DnaK binding peptide that is assumed to occupy the DnaK substrate binding groove. To this end, we used the FoldX force-field [10] to identify such potential stretches within each full peptide. Each peptide from the binders set was threaded through the DnaK binding pocket using the crystal structure of DnaK bound to a substrate peptide [5]. In this way, the binding energy of each possible heptapeptide from every full peptide was calculated with FoldX (see material and methods). The best binding heptapeptide from each full peptide was selected to fill the positive learning set of heptapeptides. To allow multiple potential heptapeptide DnaK binders per full peptide, we also added those heptapeptides with a binding energy within 0.5 kcal/mol of the binding energy of the best binding heptapeptide.

The final negative and positive learning sets contained 443 and 56 heptapeptides, respectively.

### Receiver operator characteristic (ROC) curves and Matthews correlation coefficient as predictor performance markers

The performance of the intermediate and final predictor PSSMs was assessed by *receiver operating characteristic* (ROC) curves in which, for a certain score threshold, the percentage of false positive predictions is plotted against the percentage of true positive predictions (see material and methods for detailed explanation of data point generation). The performance of each PSSM was measured against the aforementioned benchmark sets of known true positive binders and true negative binders. The calculation of ROC curves allows finding a predictor with high specificity, i.e. to find a score threshold above which the amount of false positives remains acceptable, while still finding a large amount of true positives. In this study we aim at specificity between 90–100%, i.e. 0–10% false positives. The overall performance for a certain score threshold is calculated via the Matthews correlation coefficient (MCC) (see material and methods for formula). Hence, we will use the highest MCC in the 90–100% specificity area as a predictor performance marker.

### Sequence based PSSM

Two separate PSSMs were constructed from the positive and negative learning sets using the log odd based method, similar to a recently developed amyloid prediction algorithm (Maurer-Stroh *et al*, unpublished data). For each position in the heptapeptides, the residue frequency was calculated by normalising the number of residue occurrences by the total number of sequences in each learning set. The logarithm of the ratio of the observed frequency over the expected frequency was calculated and used as the PSSM value for each position of the heptapeptide (see material and methods for formula). The expected frequency is the occurrence of residues in large databases, for which we used the Swiss-Prot database frequencies [13]. The log odd score based PSSM has advantages over the frequency based PSSM; it accounts for the chance of randomly finding a specific residue and inherently puts more weight on (DnaK) motif specific residues.

The non-binders PSSM now represents a sequence profile which is unfavorable for DnaK binding, whereas the binders PSSM reflects residue preferences for binding. To incorporate both types of data, we generated the final sequence-based PSSM by subtracting the scores of the non-binders PSSM from the scores of the binders PSSM, hereby reaching a consensus profile from the experimental data.

### Structure based PSSM

**Figure 2:**
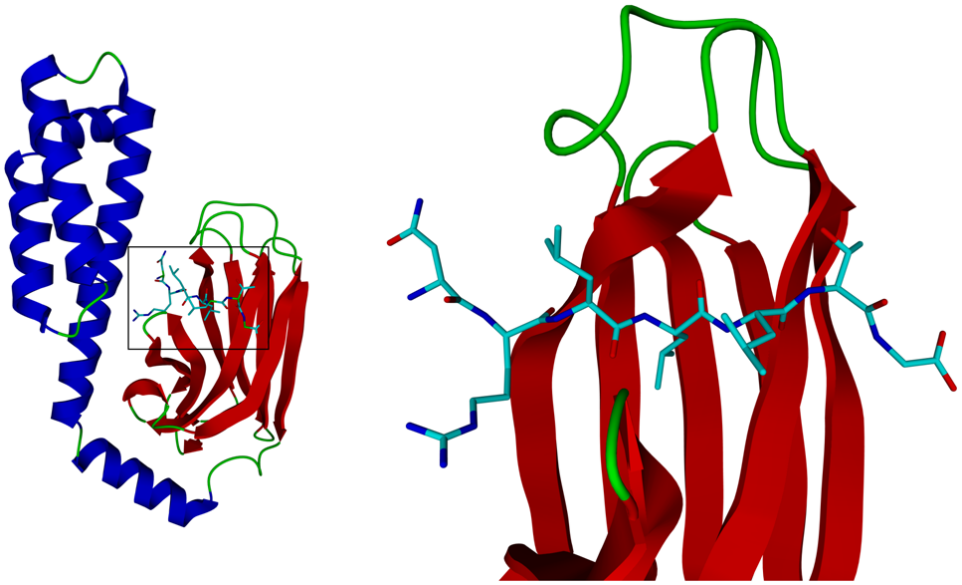
DnaK bound to a substrate peptide. Left: Substrate binding C-terminal domain of DnaK shown in cartoon style with bound heptapeptide NRLLLTG in stick style. Right: Detailed view of substrate binding in the beta sheet sandwich of DnaK. Molecular graphics created with YASARA (http://www.yasara.org) and Povray (http://www.povray.org).

To generate the structure based residue profile of DnaK substrate binding, we used the high resolution crystal structure published by Zhu *et al* as a template structure [5]. A heptapeptide with the sequence NRLLLTG, identified in a phage-display library screen [14], was co-crystallized and showed a substrate recognition motif of DnaK for a minimum of 7 residues arranged in an extended peptide conformation (Figure 2). To address the contribution of every possible amino acid at each of the seven positions in the heptapeptide, we used the FoldX force-field to perform a position scan on the heptapeptide. Firstly, the heptapeptide was mutated to poly-alanine. Next, each position was sequentially mutated to all other 19 amino acids, while the other 6 residues remained as alanine. The binding energy for each residue at every position was calculated and subtracted from the binding energy of alanine at the same position, resulting in the corresponding ΔΔG for each residue and position. The more negative the ΔΔG, the better the residue fits DnaK binding. To convert each ΔΔG into a PSSM score, we took the negative of each ΔΔG and filled the structure-based PSSM accordingly.

**Figure 3:**
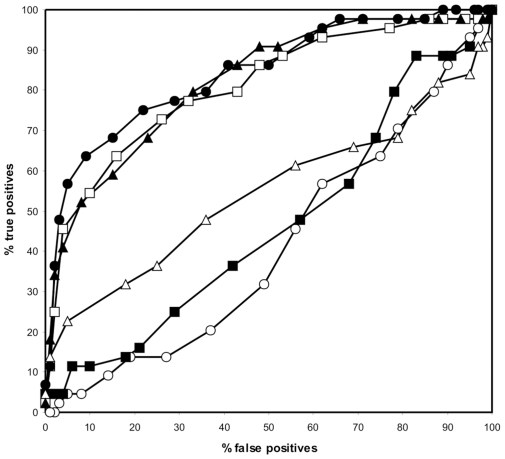
ROC curves as calculated from the PSSMs of different DnaK-substrate structures. The curves represent structures 1DKX (closed circle), 1DKY A (open square), 1DKY B (closed triangle) and 3 NMR structures from the same ensemble (open triangle, open circle, closed square).

Peptide backbone variation could influence the quality of the resulting PSSM. Therefore we assessed multiple backbone conformations of the entire structure by generating a ROC curve for PSSMs originating from different structures and calculating the MCC in the high specificity area. Structures of DnaK in complex with a substrate peptide were gathered from an NMR ensemble (PDB code 1Q5L) [15] and from different crystal structures (PDB codes 1DKX and 1DKY, of which the latter is a DnaK dimer with monomers A and B) [5]. The MCC of structure 1DKX was the highest as compared to 1DKY (A and B) and was above the MCCs of three randomly picked NMR structures from the ensemble 1Q5L (Table 1 and Figure 3). Moreover, the overall performance of the NMR structures was much lower than that of any X-ray structure, as shown in the ROC curves. Therefore we continued with the PSSM of the crystal structure 1DKX (See suppl. Table S2b for PSSM).

**Table 1:** Matthews correlation coefficient of a structure-based prediction in the high specificity area (90–100%) for different DnaK starting template structures.

Template structure	MCC at high specificity
1Q5L_1	−0.010
1Q5L_11	0.179
1Q5L_15	0.271
1DKY_A	0.512
1DKY_B	0.496
1DKX	0.588

### Performance assessment and learning set optimization

**Figure 4:**
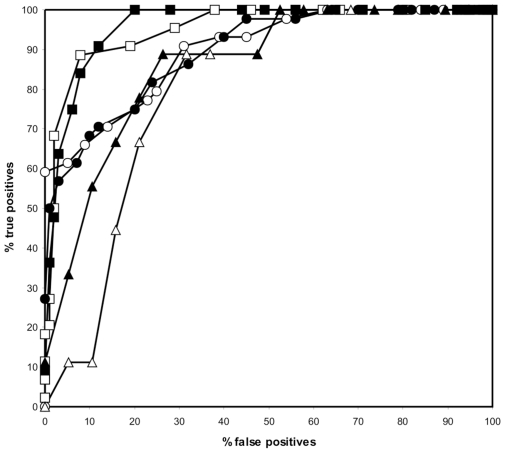
ROC curves representing the performance of the different predictors before learning set optimization. The graph visualizes the not cross-validated sequence-based predictor (open square), the not cross-validated sequence and structure-based predictor (closed square), the cross-validated sequence-based predictor (open circle), the cross-validated sequence and structure-based predictor (closed circle), the sequence based predictor on the independent validation set (open triangle) and the sequence and structure-based predictor on the independent validation set (closed triangle).

The performance of the sequence based matrix and the combination of the sequence- and structure-based matrices was also assessed by means of ROC curves. To test the robustness of the learning set, each predictor was subjected to a simulated cross-validation and a cross-validation against the validation peptide set.. During the simulated cross-validation we excluded sequences and their close homologs sequentially from the learning sets and calculated the rate of repredicting them. The MCC of the sequence-based predictor was 0.792, but this dropped to 0.708 after a simulated cross-validation assessment. While these MCC values are acceptable, the MCC of this predictor against the validation set was only 0.106, indicating that the current learning sets of peptides are not suitable to predict DnaK binding of peptides of which there is no prior knowledge in the learning sets. Next, we added the structure-based matrix scores to the sequence-based matrix scores. Before cross-validation, the MCC had a value of 0.756, reduced to 0.626 upon simulated cross-validation and 0,375 when the validation set was assessed. Although the not cross-validated and simulated cross-validation MCC had a slight performance setback (but still acceptable) upon combining sequence and structure-based PSSMs, the validation set MCC improved remarkably (Figure 4 for the ROC curves). It seems thus that adding structural information broadens the generality of the predictor.

**Figure 5:**
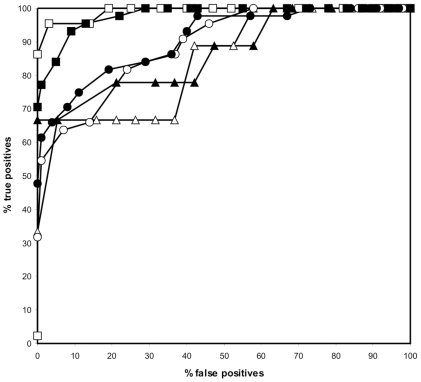
ROC curves representing the performance of the different predictors generated after running the learning set training algorithm. The graph visualizes the not cross-validated sequence-based predictor (open square), the not cross-validated sequence and structure-based predictor (closed square), the cross-validated sequence-based predictor (open circle), the cross-validated sequence and structure-based predictor (closed circle), the sequence based predictor on the independent validation set (open triangle) and the sequence and structure-based predictor on the independent validation set (closed triangle).

Although the heptapeptides in the learning sets were selected on a methodologically acceptable basis, inconsistencies in the learning set selection could not be excluded. We recently developed a training algorithm in which learning set sequences are sequentially removed and the effect on the cross-validation is assessed (Maurer-Stroh *et al*, unpublished data). Only when the Matthews correlation coefficient in the high specificity area improved, removal of the learning set sequence was accepted. The algorithm removed 8 sequences from the learning sets when the sequence-based PSSM was used to evaluate the MCC. The final not cross-validated MCC became 0.919, whereas simulated cross-validation showed an MCC of 0.651 and the validation set had an MCC of 0.662, a huge improvement compared to the starting MCC values for the sequence-based PSSM (See suppl. Table S2a for PSSM). Next, we trained the algorithm with the combined sequence- and structure-based PSSM. Here, 6 of the 499 learning set sequences were removed. The not cross-validated MCC reached 0.819 and upon cross-validation we were able to obtain an MCC of 0.703. This optimized sequence and structure based predictor had an MCC of 0.759 on the validation set sequences (Figure 5 for the ROC curves and suppl. Table S2c for PSSM)). The accuracy of the non cross-validated predictor was 92,4% in which 77,3% of the experimentally verified binding peptides were correctly predicted (true positives) with only 1% false positives (or 99% specificity). It is important to note that a higher rate of true positive detections can be achieved (93,2% instead of 77,3%) but at the cost of a lower specificity (91%). These settings can easily be customized in the online version of the predictor. When ran over the validation set, the highest obtainable accuracy was 89% with 66,7% true positives and no false positives. The performance appears to be stable when analyzing benchmark sets of variable redundancy (Suppl. Table S3, Suppl. Figure S1). The evolution of the best MCC at high specificity for the various predictors is listed in Table 2.

**Table 2:** Best Matthews correlation coefficients at high specificity (>90%) of the different predictors in the building and final stages of development.

	Benchmark set	Simulated cross-validation	Validation set
Not optimized PSSMs			
Sequence	0.792	0.708	0.106
Structure	0.588	-	0.593
Sequence+Structure	0.756	0.626	0.375
Optimized PSSMs			
Sequence	0.919	0.651	0.662
Sequence+Structure	0.819	0.703	0.759

### Residue preferences for DnaK binding

**Figure 6:**
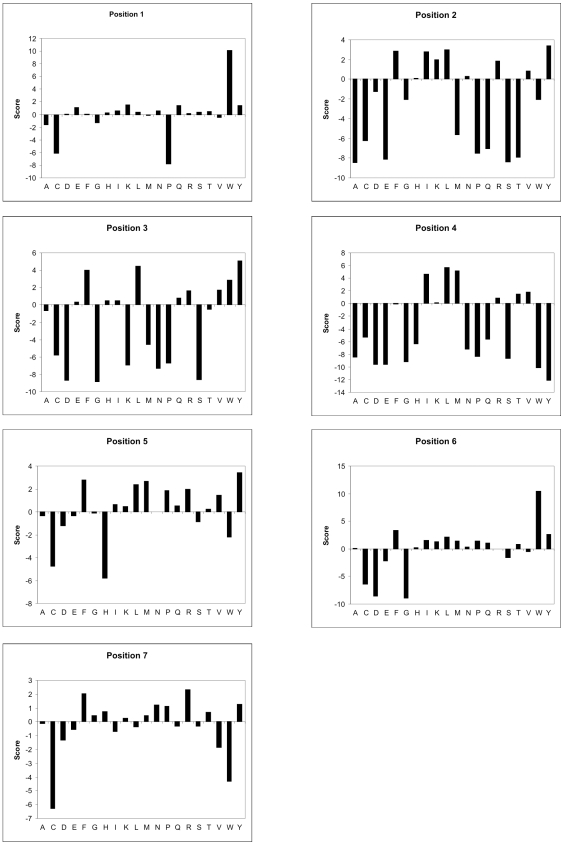
Graphical representation of the final DnaK heptameric binding profile over all aminoacids and all residue positions. The axes crossing point at score zero was chosen for convenience. Position 1 and 7 respectively represent the N-and and C-terminal end of the heptameric motif.

Our DnaK binding profile confirms previous observations of a hydrophobic core with basic flanking residues [3], albeit not as strict as once thought. We are able to define specific residue preferences and disfavors of each of the seven peptide positions (numbered N-terminally from 1 to 7). Positions 2, 3, 4, 5 and 6 score high for hydrophobic residues, with all but position 4 having also preference for the aromatic resdues F and Y (W also at position 6). The basic residue preference is most apparent at position 7, where R is the most preferred residue, after the aromatic F. Positions 2, 3 and 5 also allow basic residues (preferably R), but this feature is not as pronounced as at position 7. Position 4 is the most restricted position, where only L, I, M and to a lesser extent T and V are observed. Most other amino acids are not allowed at this central position. Positions 2 and 3 are also very restricted in their residue preference. The majority of amino acids is not observed in binding sequences at these positions. Hydrophobicity (mainly L), aromaticity (mainly F,Y) and positive charge (R) seem to be the requirements here. The large aromatic residue W is strongly preferred at the first and sixth position. Overall there seems to be an underrepresentation of C and D. E is strongly disfavoured at the second position, but shows a positive score at the very first position. A graphical representation of the profile is given in Figure 6 (See Suppl. Table S2c for PSSM).

## Discussion

From a dataset of 144 non-redundant experimentally tested peptide sequences, we were able to build a predictor for DnaK binding protein motifs by adding structural information obtained through molecular modelling. The final predictor performs well under cross-validated conditions.

The success rate was dependent on two main variables. Firstly, the training set was well optimised since the training set optimisation algorithm rectifies to a certain extent anomalies from in silico analysis. Our initial positive learning set was, in part, generated from in silico threading; from each full peptide the heptapeptide with the best binding energy was selected and also those heptapeptides with a binding energy within 0.5 kcal/mol of the heptapeptide with the best binding energy. The latter step is prone to some misclassification, since we allow multiple DnaK binding sites per peptide and this might not always be the case *in vitro*. However, our learning set training algorithm was able to remove 6 heptapeptides that appeared ‘noisy’ to allow acceptable cross-validated prediction of the benchmark peptide set. The second and most profound variable is the initial perspective of this study, that combined sequence and structural data will complement each other to detect protein-protein interaction motifs. The ROC curves in Figure 5 illustrate the power of merging both approaches: the overall performance of the dual based predictor exceeds the individual sequence- and structure-based predictors (see also Table 2). Each approach must therefore, to a certain extent, contribute information that the other lacks. The sequence learning set or alignment of both binders and non-binders must inherently provide information on the ability of seven consecutive residues to adopt the extended conformation, since this represents the DnaK binding mode of a substrate peptide. The structural approach serves to further refine the sequence information by detecting direct peptide binding preferences based on amino acid sidechain volume, charge, hydrophobicity, etc. The major improvement in the cross-validation upon adding the structural information could also be explained as the ability of the structural data to cancel out some positional residue bias or any missing residue information inside the sequence data set.

It should be noted however, that the structural template was chosen according to its stand-alone MCC and ROC performance with the experimental benchmark sequence set (Figure 3 and Table 1). Structure profiles based on NMR structures were unable to come close to the performance obtained with the X-ray structure. Moreover, the fact that out of three crystal structures the one with the highest resolution performed best should stress the use of high quality crystal structures to obtain a reliable structure-based profile.

Our dual approach DnaK profile confirmed in part the previously observed residue preferences for basic residues flanking a hydrophobic core region [3]. We extended this to show that positions 1 and 3 do not totally disfavour the negatively charged glutamate. Aspartate on the other hand is strongly rejected in all positions. The central position 4 prefers the aliphatic hydrophobic amino acids leucine, isoleucine and methionine. It is the most restricted position, which is not remarkable since the residue must fit in a well defined hydrophobic pocket of DnaK [5]. Aromatic residues, although not always evenly distributed, are very abundant at all sites, except at the central position which is neutral for phenylalanine and the last position which disfavors tryptophan. The second and third positions show a clear spectrum of aliphatic, aromatic and basic residues. The absolute last position prefers arginine, and phenylalanine (Figure 6). Overall, phenylalanine is the only residue not to have a clear disfavored site.

**Figure 7:**
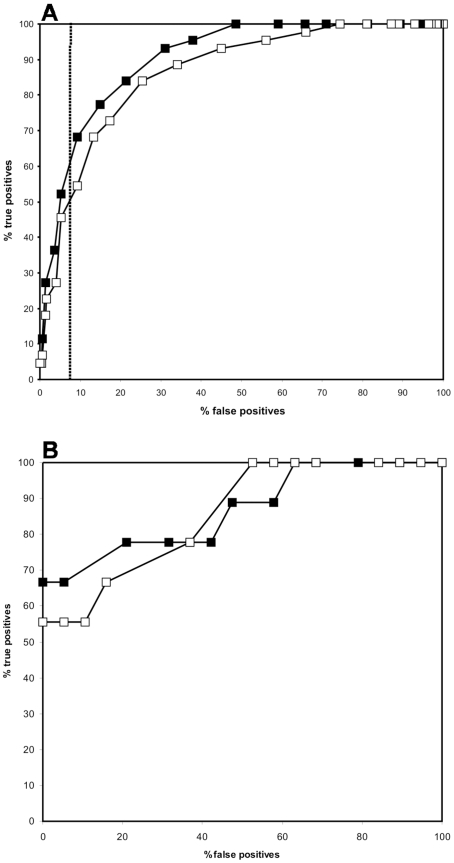
ROC performance comparison with a previously published algorithm. A: Comparison of our DnaK predictor (closed square) with the previously published predicting algorithm from Rüdiger *et al* [3] (open square) for the extended benchmark peptide set. The data point above 90% specificity (90.7%) with the best MCC in both predictors is shown as a dashed line. B: ROC curves comparison between our predictor (closed square) and the algorithm of Rüdiger *et al* (open square) for the validation set of peptides.

We have compared our method with a previously published predicting algorithm [3] (see supplementary Table S2d for PSSM). To test the generality and possible overprediction of sequences that are not known DnaK binders, we added 200 randomly selected 15-mer peptides from real proteins to the negative benchmark set. Additionally, the peptides were selected with the prerequisite to have less than 50% sequence identity to the learning set sequences. In this task, both methods perform comparably well at relevant levels of high specificity of 90.3% (Figure 7A). As can be seen in the ROC curve for this specificity, our method reaches a sensitivity of 68.2% where the previously published algorithm has a sensitivity of 54.5%. For the same amount of false positives, we predict thus more true positives compared to the algorithm of Rüdiger *et al.* in this experiment. We also compared the performance of both predictors on the validation set of peptide sequences (Figure 7B) and conclude that our method performs slightly better in terms of true positive predictions (sensitivity). Of note, the algorithm of Rüdiger *et al.* was modified from a 13-mer scoring matrix to a 7-mer scoring matrix to score heptapeptides (see supplementary Table S2d for PSSM).

In summary, we have shown that the combination of sequence and structural data can be combined to generate a validated DnaK-substrate binding predictor using experimentally tested peptide sequences. Given a well sampled sequence dataset and one or more high quality crystal structures, the same methodology can be applied to generate other protein-protein interaction predictors.

The algorithm is freely accessible at http://limbo.vib.be

## Materials and Methods

### In vitro peptide binding

For DnaK-peptide binding determination peptides where chemically sensitized on cellulose beta-alanine membranes (PepSpot membranes, JPT Peptide Technologies, GmbH). The membranes were washed in 100% MeOH for ten minutes, and three times for twenty minutes in 20 mM Tris buffer pH 7.5 plus 150 mM NaCl (TBS buffer). The membranes were blocked in blocking buffer (BSA 2% w/v in TBS buffer) for one hour. The blocked membranes were incubated in 100 nM DnaK (Axxora) in 31 mM Tris pH 7.5, 170 mM NaCl, 6.4 mM KCl, 5% Sucrose, 0.05% Tween-20 for one hour. Subsequently membranes were washed three times for ten minutes in TBS buffer and incubated for one hour with mouse monoclonal anti-DnaK antibody (clone 8E2/2, Stressgen) diluted 1∶2000 in blocking buffer plus 0.05% v/v Tween20. Membranes were washed three times for ten minutes with TBS buffer plus 0.05% v/v Tween20 and incubated for 30 minutes with anti-mouse HRP-conjugated antibody (Promega) diluted 1∶10000 in blocking buffer plus 0.05% v/v Tween20. The membranes were finally washed two times for ten minutes in TBS buffer plus 0.05% Tween20, one time for ten minutes in TBS buffer and subjected to chemiluminescent reaction using SuperSignal West Dura substrate (Pierce), detected by CCD camera connected to the ChemiDoc XRS image acquire and process system (BioRad).

### ROC curve data point generation

All benchmark peptides were scored with every intermediate and final scoring matrix as described in the results section of this article. The range between the minimum and maximum score was calculated and divided in 30 equally sized bins. Each bin threshold was compared to the score of the individual peptides. When the peptide score was above a certain threshold, we consider the peptide as a predicted binder. If the peptide was indeed an experimentally verified DnaK binder, the number of predicted true positives was increased by one. When the predicted binder was shown to be an experimentally verified non-binder, the number of predicted false positives was increased by one for that threshold. Each tuple of false positives and true positives for such a threshold becomes one data point in the ROC curve.

### Matthews correlation coefficient

The Matthews correlation coefficient (MCC) is a measure of quality of binary classifications and is given by the formula:
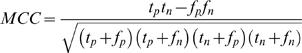
Where *t_p_ = true positives*, *t_n_ = true negatives*, *f_p_ = false positives*, *f_n_ = false negatives*. An MCC value of 1 is for a perfect prediction, 0 for a completely random assignment and −1 for the worst possible prediction.

### Position specific scoring matrix (PSSM) calculation

For both the alignments of the binding and non-binding heptapeptides the number of observed residue occurrences *n_obs_* at each position was counted. Next, this value was normalised by the number of aligned sequences in each learning set, which resulted in the positional residue frequency *f_obs_*:
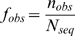
The final matrix score *S* was calculated by taking the logarithm of the ratio of the observed frequency *f_obs_* over the experimental (database) residue frequency *f_ex_*:
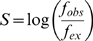
Some residues do not occur at all at certain positions in the heptapeptide alignment which leads to the logarithm of zero. To circumvent this issue, we implemented so-called pseudocounts: whenever a zero count occurred, it was substituted by 0.001.

### *In silico b*inding energy calculations using FoldX

We employed FoldX version 2.7 to model mutants of the peptide bound to DnaK in the crystal structure (PDB code: 1DKX). To this end, the peptide was first reduced to poly-Alanine. Then, all possible natural amino acids were systematically introduced at each position, while keeping the remainder of the peptide as alanines. Energy estimates were calculated with FoldX as the ΔG difference (ΔΔG) to the reference poly-Alanine. This method reduces the sequence space to be covered by the modelling dramatically, but ignores any dependencies between the positions. Given the extended conformation of the peptide bound to DnaK, this assumption seems reasonable.

## Supporting Information

Table S1 Full list of peptides tested for DnaK binding by means of cellulose-based scans
(0.36 MB DOC)

Table S2 The various position specific scoring matrices generated and used in the manuscript
(0.14 MB DOC)

Table S3 Performance analysis of the predictor on benchmark sets with varying degrees of redundancy
(0.04 MB DOC)

Figure S1 ROC curves to evaluate the performance of the algorithm on benchmark sets with varying degree of redundancy.
(0.04 MB DOC)

## References

[1] Mayer MP, Bukau B (2005). Hsp70 chaperones: cellular functions and molecular mechanism.. Cell Mol Life Sci.

[2] Hartl FU, Hayer-Hartl M (2002). Molecular chaperones in the cytosol: from nascent chain to folded protein.. Science.

[3] Rudiger S, Germeroth L, Schneider-Mergener J, Bukau B (1997). Substrate specificity of the DnaK chaperone determined by screening cellulose-bound peptide libraries.. EMBO J.

[4] Flaherty KM, McKay DB, Kabsch W, Holmes KC (1991). Similarity of the three-dimensional structures of actin and the ATPase fragment of a 70-kDa heat shock cognate protein.. Proc Natl Acad Sci U S A.

[5] Zhu X, Zhao X, Burkholder WF, Gragerov A, Ogata CM (1996). Structural analysis of substrate binding by the molecular chaperone DnaK.. Science.

[6] Szabo A, Langer T, Schroder H, Flanagan J, Bukau B (1994). The ATP hydrolysis-dependent reaction cycle of the Escherichia coli Hsp70 system DnaK, DnaJ, and GrpE.. Proc Natl Acad Sci U S A.

[7] Rudiger S, Schneider-Mergener J, Bukau B (2001). Its substrate specificity characterizes the DnaJ co-chaperone as a scanning factor for the DnaK chaperone.. EMBO J.

[8] Antes I, Siu SW, Lengauer T (2006). DynaPred: a structure and sequence based method for the prediction of MHC class I binding peptide sequences and conformations.. Bioinformatics.

[9] Brannetti B, Via A, Cestra G, Cesareni G, Helmer-Citterich M (2000). SH3-SPOT: an algorithm to predict preferred ligands to different members of the SH3 gene family.. J Mol Biol.

[10] Schymkowitz J, Borg J, Stricher F, Nys R, Rousseau F (2005). The FoldX web server: an online force field.. Nucleic Acids Res.

[11] Fernandez-Escamilla AM, Rousseau F, Schymkowitz J, Serrano L (2004). Prediction of sequence-dependent and mutational effects on the aggregation of peptides and proteins.. Nat Biotechnol.

[12] McCarty JS, Rudiger S, Schonfeld HJ, Schneider-Mergener J, Nakahigashi K (1996). Regulatory region C of the E. coli heat shock transcription factor, sigma32, constitutes a DnaK binding site and is conserved among eubacteria.. J Mol Biol.

[13] Boeckmann B, Bairoch A, Apweiler R, Blatter MC, Estreicher A (2003). The SWISS-PROT protein knowledgebase and its supplement TrEMBL in 2003.. Nucleic Acids Res.

[14] Gragerov A, Zeng L, Zhao X, Burkholder W, Gottesman ME (1994). Specificity of DnaK-peptide binding.. J Mol Biol.

[15] Stevens SY, Cai S, Pellecchia M, Zuiderweg ER (2003). The solution structure of the bacterial HSP70 chaperone protein domain DnaK(393–507) in complex with the peptide NRLLLTG.. Protein Sci.

