# Supplementary material for: Accurate Prediction of DnaK-Peptide Binding via Homology Modelling and Experimental Data
Source: PLoS Comput Biol. 2009 Aug 21;5(8):e1000475. doi: 10.1371/journal.pcbi.1000475 (PMC2717214; doi:10.1371/journal.pcbi.1000475)
Supplement: Table S1 — Full list of peptides tested for DnaK binding by means of cellulose-based scans (0.36 MB DOC) [file pcbi.1000475.s001.doc]

**Table S1**: Full list of peptides tested for DnaK binding by means of cellulose-based scans.

| Sequence identifier | Sequence | Raw data | | source |
| --- | --- | --- | --- | --- |
| Group 1 (Membrane A) | | | | |
| Membrane A |  |  | |  |
| tango_amorf_884 | LSLLLLFALWIRIG | 25162,02269 | | Q9Y3K6 |
| tango_amorf_845 | KYLFTFVMVIPETM | 38855,28528 | | Q96K04 |
| tango_amorf_840 | VPVLLSLLLLLGPA | 42710,84545 | | P25311 |
| tango_amorf_832 | LFVWFLYSLLMKLF | 11753,93187 | | P00387 |
| tango_amorf_830 | GEEIIIKIFFIDPN | 38062,92551 | | O95619 |
| tango_amorf_708 | GRFGVYLLYWLNPR | 24274,81664 | | Q9H5H2 |
| tango_amorf_705 | VKVLITLYWLGRKA | 34738,01627 | | Q8WWI1 |
| tango_amorf_689 | MRFIAAFFTIAKTR | 58125,53643 | | Q8N933 |
| tango_amorf_618 | EFQAVVMAVGGGSR | 3753,616245 | | Q9NR50 |
| tango_amorf_597 | KQGEILGVVIVESG | 3190,86766 | | Q02410 |
| tango_amorf_559 | VRSITLFIINLHRS | 22773,05368 | | Q9HB39 |
| tango_amorf_525 | ANALAVLAVG | 10596,74392 | | P0A9D0 |
| tango_amorf_485 | QHVEKVVFWLHDSF | 10242,53962 | | Q03111 |
| tango_amorf_472 | AFPLFALVWGLNL | 13819,5284 | | P22709 |
| tango_amorf_465 | KEFLLIMRELLDPK | 23838,65032 | | Q9HCH9 |
| tango_amorf_423 | TELYFVLDFVNGGE | 32297,64582 | | Q96BR1 |
| tango_amorf_397 | LQLSSILLMFSNPT | 66870,96952 | | Q96Q95 |
| tango_amorf_384 | VPDIAGVLAVYAERR | 9366,997215 | | Q9XB42 |
| tango_amorf_372 | AALLAVSLLP | 26799,27388 | | P21338 |
| tango_amorf_357 | GLDGFITITGGKLM | 3581,78082 | | P0A9C1 |
| tango_amorf_340 | QEVISLGVLGIRSP | 9016,228716 | | Q96M46 |
| tango_amorf_317 | FAFFSIGVQG | 57779,33394 | | P76396 |
| tango_amorf_257 | RMELMAAIVALEAL | 4658,49861 | | P0A7Y4 |
| tango_amorf_255 | SEIDVIAVTKGRGV | 3232,639807 | | Q92901 |
| tango_amorf_240 | YSDGLFSFSVNVNRA | 22946,10972 | | P0AFX9 |
| tango_amorf_118 | SPRAYLVYNATDTV | 14035,17033 | | P17315 |
| tango_amorf_104 | LTKMMTSYVIGQAM | 5574,140467 | | P0AEB2 |
| tango_amorf_91 | ADVISAFGSVLSDP | 2993,399326 | | Q12882 |
| tango_amorf_70 | MALVNLSAVA | 2848,553048 | | P0AGG8 |
| tango_amorf_56 | NQKLIVLGNLTVKGN | 36546,15351 | | P25907 |
| tango_amorf_45 | SDMIVAGTLTNKMA | 1056,555043 | | Q9H3K5 |
| tango_amorf_39 | SHVGLLTTLNFGDG | 7569,619464 | | Q8WVV8 |
| tango_amorf_38 | VIAMSAAVGL | 1892,856945 | | P0ADF6 |
| tango_amorf_17 | MATGIAVQIL | 2810,533161 | | P75859 |
| tango_amorf_14 | LKLQQFAACFLETL | 4755,062795 | | Q13111 |
| tango_amorf_13 | LQRVAVGAALLSMP | 6546,066225 | | P0A940 |
| tango_amorf_11 | SNDLTQYLLAVDRD | 11981,59911 | | P32670 |
|  |  |  | |  |
| Group 1 (Membrane B) | | | | |
| alkfoswt | EGNTLVIVTADHAAA | 9985,780298 | | P00634 |
| Elkfos | EGNELVIVTADHAAA | 4660,860139 | | P00634 mutant |
| alkfoE | EGNTLVIVEADHAAA | 5713,58017 | | P00634 mutant |
| alEfos | EGNTLVEVTADHAAA | 705,320021 | | P00634 mutant |
| ElkfoE | EGNELVIVEADHAAA | 2026,37006 | | P00634 mutant |
| Dlkfos | EGNDLVIVTADHAAA | 3251,170097 | | P00634 mutant |
| alkfoD | EGNTLVIVDADHAAA | 4866,840145 | | P00634 mutant |
| alDfos | EGNTLVDVTADHAAA | 488,5300146 | | P00634 mutant |
| DlkfoD | EGNDLVIVDADHAAA | 1086,270032 | | P00634 mutant |
| Rlkfos | EGNRLVIVTADHAAA | 4798,910143 | | P00634 mutant |
| alkfoR | EGNTLVIVRADHAAA | 8998,920268 | | P00634 mutant |
| alRfos | EGNTLVRVTADHAAA | 2786,600083 | | P00634 mutant |
| RlkfoR | EGNRLVIVRADHAAA | 7910,480236 | | P00634 mutant |
| Klkfos | EGNKLVIVTADHAAA | 4839,880144 | | P00634 mutant |
| alkfoK | EGNTLVIVKADHAAA | 6137,400183 | | P00634 mutant |
| alKfos | EGNTLVKVTADHAAA | 2166,890065 | | P00634 mutant |
| KlkfoK | EGNKLVIVKADHAAA | 9010,410269 | | P00634 mutant |
| Plkfos | EGNPLVIVTADHAAA | 6573,690196 | | P00634 mutant |
| alkfoP | EGNTLVIVPADHAAA | 3942,770118 | | P00634 mutant |
| alPfos | EGNTLVPVTADHAAA | 819,8900244 | | P00634 mutant |
| PlkfoP | EGNPLVIVPADHAAA | 5881,300175 | | P00634 mutant |
| sucK | PEPHILLFKREKDAA | 18008,28054 | | P08463 |
| sucR | PEPHILLFRREKDAA | 12752,66038 | | P08463 mutant |
| sucP | PEPHILLFPREKDAA | 5998,620179 | | P08463 mutant |
| sucD | PEPHILLFDREKDAA | 786,0900234 | | P08463 mutant |
| sucE | PEPHILLFEREKDAA | 2856,120085 | | P08463 mutant |
| sucH | PEPHILLFHREKDAA | 2360,09007 | | P08463 mutant |
| sucQ | PEPHILLFQREKDAA | 9186,100274 | | P08463 mutant |
| sucN | PEPHILLFNREKDAA | 3456,190103 | | P08463 mutant |
| sucG | PEPHILLFGREKDAA | 3035,56009 | | P08463 mutant |
| sucS | PEPHILLFSREKDAA | 8136,250242 | | P08463 mutant |
| sucC | PEPHILLFCREKDAA | 745,6300222 | | P08463 mutant |
| sucT | PEPHILLFTREKDAA | 9377,790279 | | P08463 mutant |
| sucA | PEPHILLFAREKDAA | 8000,610238 | | P08463 mutant |
| sucM | PEPHILLFMREKDAA | 4420,050132 | | P08463 mutant |
| sucY | PEPHILLFYREKDAA | 8657,430258 | | P08463 mutant |
| sucW | PEPHILLFWREKDAA | 4188,940125 | | P08463 mutant |
| sucL | PEPHILLFLREKDAA | 15754,95047 | | P08463 mutant |
| sucI | PEPHILLFIREKDAA | 13087,39039 | | P08463 mutant |
| sucV | WGGPEPHILLFVREKDAA | 9082,750271 | | P08463 mutant |
| sucF | PEPHILLFFREKDAA | 13816,34041 | | P08463 mutant |
| Abtail | QKLVFFAEDVAA | 10912,56033 | | P05067 |
| Manoli | STVIIEAA | 458,7200137 | | Synthetic [1] |
| tango1 | STLIVLAA | 28208,85084 | | Synthetic [1] |
| tango2 | KTVIIEAA | 675,0600201 | | Synthetic [1] |
| tango3 | STVIFEAA | 672,45002 | | Synthetic [1] |
| sup35 | NNQQNYQAA | 619,8300185 | | P05453 |
| b2micro20-41 | SNFLNCYVSFHPSDIEVDLLKAA | 836,5200249 | | P61769 |
| b2micro59-71 | DWSFYLLYYTEFTPTGKDEYAAA | 6795,800203 | | P61769 |
| b2micro83-89 | NHVTLSQAA | 1302,960039 | | P61769 |
| transthyretin10-19 | PLMVKVLDAAA | 22288,83066 | | P02766 |
| transthyretin105-115 | YTIAALLSPYSAA | 10615,16032 | | P02766 |
| lysozyme49-64 | STDYGILQINSRWWCAA | 597,8600178 | | P00698 |
| apolipoA1_52-57 | SVTSTFAA | 742,8400221 | | P02647 |
| calcitonin15-19 | DFNKFAA | 325,0100097 | | P01258 |
| ab15-24 | KLVFFAEAA | 13107,86039 | | P05067 |
| isletamyloid20-29 | SNNFGAILSSAA | 833,6000248 | | P10997 |
| isletamyloidbis | NFLVHAA | 1417,580042 | | P10997 |
| Medin286-293 | NFGSVQFVAA | 911,1000272 | | Q08431 |
| alphasynnuclein68-78 | GAVVTGVTAVAQKTVEGAAA | 2743,440082 | | P37840 |
| PrP106-126 | KTNMKHMAGAAAAGAVVGGLGAA | 454,4100135 | | P04156 |
| PrP127-147 | YMLGSAMSRPIIHFGSDYEDAA | 580,1200173 | | P04156 |
| PrP178-193 | DCVNITIKQHTVTTTTAA | 1610,970048 | | P04156 |
| sup35_7-13 | GNNQQNYAA | 539,4700161 | | P05453 |
| DNAKbinder1 | GNRLLTGAA | 6957,480207 | | Synthetic [2] |
| DNAKbinder2 | GRLLLLLGAA | 20135,1506 | | Synthetic |
|  |  |  | |  |
| Group 1 (Membrane C) | | | | |
| SigmaPD | AMAPVLYLQDKSS | 52,64442776 | | P0AGB3 |
| SigmaSD | AMASVLYLQDKSS | 52,50756191 | | P0AGB3 mutant |
| SigmaPS | AMAPVLYLQSKSS | 76,01752316 | | P0AGB3 mutant |
| SigmaSS | AMASVLYLQSKSS | 81,61973352 | | P0AGB3 mutant |
| SigmaPP | AMAPVLYLQPKSS | 68,80719066 | | P0AGB3 mutant |
| SigmaRD | AMARVLYLQDKSS | 80,17779088 | | P0AGB3 mutant |
| SigmaPR | AMAPVLYLQRKSS | 120,5414502 | | P0AGB3 mutant |
| SigmaRR | AMARVLYLQRKSS | 189,9311981 | | P0AGB3 mutant |
| SigmaKD | AMAKVLYLQDKSS | 63,58172266 | | P0AGB3 mutant |
| SigmaPK | AMAPVLYLQKKSS | 82,36227721 | | P0AGB3 mutant |
| SigmaKK | AMAKVLYLQKKSS | 148,5774805 | | P0AGB3 mutant |
| SigmaDD | AMADVLYLQDKSS | 47,29386065 | | P0AGB3 mutant |
| SigmaED | AMAEVLYLQDKSS | 39,70079921 | | P0AGB3 mutant |
| SigmaPE | AMAPVLYLQEKSS | 43,70397053 | | P0AGB3 mutant |
| SigmaEE | AMAEVLYLQEKSS | 45,70122107 | | P0AGB3 mutant |
| HemaEE | YNAELLVLLENER | 149,4661796 | | Q67039 |
| HemaSE | YNASLLVLLENER | 371,9906486 | | Q67039 mutant |
| HemaES | YNAELLVLLSNER | 287,9277667 | | Q67039 mutant |
| HemaSS | YNASLLVLLSNER | 319,602612 | | Q67039 mutant |
| HemaDE | YNADLLVLLENER | 162,0161382 | | Q67039 mutant |
| HemaED | YNAELLVLLDNER | 134,2311318 | | Q67039 mutant |
| HemaDD | YNADLLVLLDNER | 111,9381 | | Q67039 mutant |
| HemaPE | YNAPLLVLLENER | 108,1341376 | | Q67039 mutant |
| HemaEP | YNAELLVLLPNER | 126,9937564 | | Q67039 mutant |
| HemaPP | YNAPLLVLLPNER | 157,8059134 | | Q67039 mutant |
| Hema RE | YNARLLVLLENER | 179,4819135 | | Q67039 mutant |
| HemaER | YNAELLVLLRNER | 265,0558935 | | Q67039 mutant |
| HemaRR | YNARLLVLLRNER | 231,6668188 | | Q67039 mutant |
| HemaKE | YNAKLLVLLENER | 224,0766475 | | Q67039 mutant |
| HemaEK | YNAELLVLLKNER | 245,3707444 | | Q67039 mutant |
| HemaKK | YNAKLLVLLKNER | 231,1883044 | | Q67039 mutant |
|  |  |  | |  |
| Group 2 (Membrane A) | | | | |
| P0A984|CSPH_ECO57_1 | MSRKMTGIVK | 4710,4 | CSPH_ECO57 | |
| P0A984|CSPH_ECO57_6 | TGIVKTFDRK | 19508,9 | CSPH_ECO57 | |
| P0A984|CSPH_ECO57_11 | TFDRKSGKGF | 9129,52 | CSPH_ECO57 | |
| P0A984|CSPH_ECO57_16 | SGKGFIIPSD | 2171,29 | CSPH_ECO57 | |
| P0A984|CSPH_ECO57_21 | IIPSDGRKEV | 3033,23 | CSPH_ECO57 | |
| P0A984|CSPH_ECO57_26 | GRKEVQVHIS | 11078 | CSPH_ECO57 | |
| P0A984|CSPH_ECO57_31 | QVHISAFTPR | 31312,2 | CSPH_ECO57 | |
| P0A984|CSPH_ECO57_36 | AFTPRDAEVL | 1633,45 | CSPH_ECO57 | |
| P0A984|CSPH_ECO57_41 | DAEVLIPGLR | 1291,82 | CSPH_ECO57 | |
| P0A984|CSPH_ECO57_46 | IPGLRVEFCR | 6854,66 | CSPH_ECO57 | |
| P0A984|CSPH_ECO57_50 | RVEFCRVNGL | 3835,04 | CSPH_ECO57 | |
| P0A984|CSPH_ECO57_56 | VNGLRGPTAA | 3248,51 | CSPH_ECO57 | |
| P0A984|CSPH_ECO57_61 | GPTAANVYLS | 1756,06 | CSPH_ECO57 | |
| P69224|IF1_ECO57_1 | MAKEDNIEMQ | 1356,46 | IF1_ECO57 | |
| P69224|IF1_ECO57_6 | NIEMQGTVLE | 826,31 | IF1_ECO57 | |
| P69224|IF1_ECO57_11 | GTVLETLPNT | 1085,17 | IF1_ECO57 | |
| P69224|IF1_ECO57_16 | TLPNTMFRVE | 993,4 | IF1_ECO57 | |
| P69224|IF1_ECO57_21 | MFRVELENGH | 844,3 | IF1_ECO57 | |
| P69224|IF1_ECO57_26 | LENGHVVTAH | 1004,75 | IF1_ECO57 | |
| P69224|IF1_ECO57_31 | VVTAHISGKM | 5156,78 | IF1_ECO57 | |
| P69224|IF1_ECO57_36 | ISGKMRKNYI | 5511,03 | IF1_ECO57 | |
| P69224|IF1_ECO57_40 | MRKNYIRILT | 18361,9 | IF1_ECO57 | |
| P69224|IF1_ECO57_46 | RILTGDKVTV | 4601,72 | IF1_ECO57 | |
| P69224|IF1_ECO57_51 | DKVTVELTPY | 2227,49 | IF1_ECO57 | |
| P69224|IF1_ECO57_56 | ELTPYDLSKG | 3690,01 | IF1_ECO57 | |
| P69224|IF1_ECO57_60 | LSKGRIVFRS | 42774,9 | IF1_ECO57 | |
| P0A9L7|PPIC_ECO57_1 | MAKTAAALHI | 3034,72 | PPIC_ECO57 | |
| P0A9L7|PPIC_ECO57_6 | AALHILVKEE | 3271,34 | PPIC_ECO57 | |
| P0A9L7|PPIC_ECO57_11 | LVKEEKLALD | 1535,99 | PPIC_ECO57 | |
| P0A9L7|PPIC_ECO57_16 | KLALDLLEQI | 5078,75 | PPIC_ECO57 | |
| P0A9L7|PPIC_ECO57_21 | LLEQIKNGAD | 1855,02 | PPIC_ECO57 | |
| P0A9L7|PPIC_ECO57_26 | KNGADFGKLA | 3249,46 | PPIC_ECO57 | |
| P0A9L7|PPIC_ECO57_31 | FGKLAKKHSI | 20557,5 | PPIC_ECO57 | |
| P0A9L7|PPIC_ECO57_36 | KKHSICPSGK | 6196,02 | PPIC_ECO57 | |
| P0A9L7|PPIC_ECO57_41 | CPSGKRGGDL | 4048,55 | PPIC_ECO57 | |
| P0A9L7|PPIC_ECO57_46 | RGGDLGEFRQ | 2121,56 | PPIC_ECO57 | |
| P0A9L7|PPIC_ECO57_51 | GEFRQGQMVP | 1608,59 | PPIC_ECO57 | |
| P0A9L7|PPIC_ECO57_56 | GQMVPAFDKV | 1448,78 | PPIC_ECO57 | |
| P0A9L7|PPIC_ECO57_62 | FDKVVFSCPV | 6400,81 | PPIC_ECO57 | |
| P0A9L7|PPIC_ECO57_66 | VFSCPVLEPT | 1242,99 | PPIC_ECO57 | |
| P0A9L7|PPIC_ECO57_71 | VLEPTGPLHT | 891,91 | PPIC_ECO57 | |
| P0A9L7|PPIC_ECO57_76 | GPLHTQFGYH | 10383,8 | PPIC_ECO57 | |
| P0A9L7|PPIC_ECO57_84 | YHIIKVLYRN | 27813,2 | PPIC_ECO57 | |
| P0A6G1|CH10_ECO57_1 | MNIRPLHDRV | 7820,03 | CH10_ECO57 | |
| P0A6G1|CH10_ECO57_6 | LHDRVIVKRK | 29702,2 | CH10_ECO57 | |
| P0A6G1|CH10_ECO57_11 | IVKRKEVETK | 13634,6 | CH10_ECO57 | |
| P0A6G1|CH10_ECO57_16 | EVETKSAGGI | 2627,35 | CH10_ECO57 | |
| P0A6G1|CH10_ECO57_21 | SAGGIVLTGS | 2146,29 | CH10_ECO57 | |
| P0A6G1|CH10_ECO57_26 | VLTGSAAAKS | 2750,63 | CH10_ECO57 | |
| P0A6G1|CH10_ECO57_31 | AAAKSTRGEV | 1868,63 | CH10_ECO57 | |
| P0A6G1|CH10_ECO57_36 | TRGEVLAVGN | 2245,3 | CH10_ECO57 | |
| P0A6G1|CH10_ECO57_41 | LAVGNGRILE | 1897,6 | CH10_ECO57 | |
| P0A6G1|CH10_ECO57_46 | GRILENGEVK | 3261,53 | CH10_ECO57 | |
| P0A6G1|CH10_ECO57_51 | NGEVKPLDVK | 3090,42 | CH10_ECO57 | |
| P0A6G1|CH10_ECO57_56 | PLDVKVGDIV | 1666,91 | CH10_ECO57 | |
| P0A6G1|CH10_ECO57_61 | VGDIVIFNDG | 9362,34 | CH10_ECO57 | |
| P0A6G1|CH10_ECO57_66 | IFNDGYGVKS | 2403,66 | CH10_ECO57 | |
| P0A6G1|CH10_ECO57_71 | YGVKSEKIDN | 1384,76 | CH10_ECO57 | |
| P0A6G1|CH10_ECO57_76 | EKIDNEEVLI | 724,37 | CH10_ECO57 | |
| P0A6G1|CH10_ECO57_81 | EEVLIMSESD | 808,18 | CH10_ECO57 | |
| P0A6G1|CH10_ECO57_86 | MSESDILAIV | 2079,25 | CH10_ECO57 | |
| Q8XAG2|URE3_ECO57_1 | MELTPREKDK | 1469,76 | URE3_ECO57 | |
| Q8XAG2|URE3_ECO57_6 | REKDKLLLFT | 25808,9 | URE3_ECO57 | |
| Q8XAG2|URE3_ECO57_10 | KLLLFTAALL | 38633,9 | URE3_ECO57 | |
| Q8XAG2|URE3_ECO57_16 | AALLAERRLA | 12208,6 | URE3_ECO57 | |
| Q8XAG2|URE3_ECO57_21 | ERRLARGLKL | 37245,6 | URE3_ECO57 | |
| Q8XAG2|URE3_ECO57_26 | RGLKLNYPES | 14345,6 | URE3_ECO57 | |
| Q8XAG2|URE3_ECO57_31 | NYPESVALIS | 3351,76 | URE3_ECO57 | |
| Q8XAG2|URE3_ECO57_36 | VALISAFIME | 5918,58 | URE3_ECO57 | |
| Q8XAG2|URE3_ECO57_41 | AFIMEGARDG | 1011,44 | URE3_ECO57 | |
| Q8XAG2|URE3_ECO57_46 | GARDGKSVAA | 1775,5 | URE3_ECO57 | |
| Q8XAG2|URE3_ECO57_51 | KSVAALMEEG | 1405,15 | URE3_ECO57 | |
| Q8XAG2|URE3_ECO57_56 | LMEEGRHVLS | 1943,44 | URE3_ECO57 | |
| Q8XAG2|URE3_ECO57_60 | GRHVLSREQV | 7349,05 | URE3_ECO57 | |
| Q8XAG2|URE3_ECO57_66 | REQVMEGIPE | 1534,09 | URE3_ECO57 | |
| Q8XAG2|URE3_ECO57_71 | EGIPEMIPDI | 1137,34 | URE3_ECO57 | |
| Q8XAG2|URE3_ECO57_76 | MIPDIQVEAT | 1022,02 | URE3_ECO57 | |
| Q8XAG2|URE3_ECO57_81 | QVEATFPDGS | 1177,53 | URE3_ECO57 | |
| Q8XAG2|URE3_ECO57_86 | FPDGSKLVTV | 1971,65 | URE3_ECO57 | |
| Q8XAG2|URE3_ECO57_91 | KLVTVHNPII | 17096,4 | URE3_ECO57 | |
| bukau1 | AKTLILSHLRFVVGAASGAA | 62212,7 | synthetic | |
| bukau2 | VVHIARNYAGYGGAASGAA | 12445,9 | synthetic | |
|  |  |  |  | |
| Group 2 (Membrane B) | | | | |
| P58233|CEST_ECO57_1 | MSSRSELLLE | 1548,146312 | CEST_ECO57 | |
| P58233|CEST_ECO57_6 | ELLLEKFAEK | 8366,047952 | CEST_ECO57 | |
| P58233|CEST_ECO57_11 | KFAEKIGIGS | 6427,033688 | CEST_ECO57 | |
| P58233|CEST_ECO57_16 | IGIGSISFNE | 15730,83019 | CEST_ECO57 | |
| P58233|CEST_ECO57_21 | ISFNENRLCS | 2889,375829 | CEST_ECO57 | |
| P58233|CEST_ECO57_26 | NRLCSFAIDE | 9717,494466 | CEST_ECO57 | |
| P58233|CEST_ECO57_31 | FAIDEIYYIS | 21610,39558 | CEST_ECO57 | |
| P58233|CEST_ECO57_36 | IYYISLSDAN | 13863,47052 | CEST_ECO57 | |
| P58233|CEST_ECO57_41 | LSDANDEYMM | 1902,847903 | CEST_ECO57 | |
| P58233|CEST_ECO57_46 | DEYMMIYGVC | 3725,948978 | CEST_ECO57 | |
| P58233|CEST_ECO57_51 | IYGVCGKFPT | 8611,391507 | CEST_ECO57 | |
| P58233|CEST_ECO57_56 | GKFPTDNSNF | 4143,580038 | CEST_ECO57 | |
| P58233|CEST_ECO57_61 | DNSNFALEIL | 20689,96168 | CEST_ECO57 | |
| P58233|CEST_ECO57_66 | ALEILNANLW | 9666,545104 | CEST_ECO57 | |
| P58233|CEST_ECO57_71 | NANLWFAENG | 14848,597 | CEST_ECO57 | |
| P58233|CEST_ECO57_76 | FAENGGPYLC | 2952,26009 | CEST_ECO57 | |
| P58233|CEST_ECO57_81 | GPYLCYEAGA | 4473,733711 | CEST_ECO57 | |
| P58233|CEST_ECO57_86 | YEAGAQSLLL | 4314,059225 | CEST_ECO57 | |
| P58233|CEST_ECO57_91 | QSLLLALRFP | 39867,22007 | CEST_ECO57 | |
| P58233|CEST_ECO57_96 | ALRFPLDDAT | 8360,170918 | CEST_ECO57 | |
| P58233|CEST_ECO57_101 | LDDATPEKLE | 1393,806365 | CEST_ECO57 | |
| P58233|CEST_ECO57_106 | PEKLENEIEV | 1600,858784 | CEST_ECO57 | |
| P58233|CEST_ECO57_111 | NEIEVVVKSM | 2769,077469 | CEST_ECO57 | |
| P58233|CEST_ECO57_116 | VVKSMENLYL | 4801,943441 | CEST_ECO57 | |
| P58233|CEST_ECO57_121 | ENLYLVLHNQ | 21014,78081 | CEST_ECO57 | |
| P58233|CEST_ECO57_126 | VLHNQGITLE | 3326,4011 | CEST_ECO57 | |
| P58233|CEST_ECO57_131 | GITLENEHMK | 3159,538549 | CEST_ECO57 | |
| P58233|CEST_ECO57_136 | NEHMKIEEIS | 1800,723139 | CEST_ECO57 | |
| P58233|CEST_ECO57_141 | IEEISSSDNK | 2194,755648 | CEST_ECO57 | |
| P58233|CEST_ECO57_146 | SSDNKHYYAG | 4334,041139 | CEST_ECO57 | |
| P67090|USPD_ECO57_1 | MAYKHIGVAI | 7136,527285 | USPD_ECO57 | |
| P67090|USPD_ECO57_6 | IGVAISGNEE | 2634,719436 | USPD_ECO57 | |
| P67090|USPD_ECO57_11 | SGNEEDALLV | 2687,657948 | USPD_ECO57 | |
| P67090|USPD_ECO57_16 | DALLVNKALE | 5702,666675 | USPD_ECO57 | |
| P67090|USPD_ECO57_21 | NKALELARHN | 16303,20806 | USPD_ECO57 | |
| P67090|USPD_ECO57_26 | LARHNDAHLT | 3715,053862 | USPD_ECO57 | |
| P67090|USPD_ECO57_31 | DAHLTLIHID | 8991,816422 | USPD_ECO57 | |
| P67090|USPD_ECO57_36 | LIHIDDGLSE | 3704,339577 | USPD_ECO57 | |
| P67090|USPD_ECO57_43 | LSELYPGIYF | 14133,49762 | USPD_ECO57 | |
| P67090|USPD_ECO57_46 | LYPGIYFPAT | 12389,55567 | USPD_ECO57 | |
| P67090|USPD_ECO57_51 | YFPATEDILQ | 4595,930805 | USPD_ECO57 | |
| P67090|USPD_ECO57_56 | EDILQLLKNK | 14505,37823 | USPD_ECO57 | |
| P67090|USPD_ECO57_61 | LLKNKSDNKL | 19091,77016 | USPD_ECO57 | |
| P67090|USPD_ECO57_68 | NKLYKLTKNI | 39589,68846 | USPD_ECO57 | |
| P67090|USPD_ECO57_71 | YKLTKNIQWP | 30076,80518 | USPD_ECO57 | |
| P67090|USPD_ECO57_76 | NIQWPKTKLR | 32551,1268 | USPD_ECO57 | |
| P67090|USPD_ECO57_81 | KTKLRIERGE | 36720,88225 | USPD_ECO57 | |
| P67090|USPD_ECO57_86 | IERGEMPETL | 2711,798994 | USPD_ECO57 | |
| P67090|USPD_ECO57_91 | MPETLLEIMQ | 2237,884034 | USPD_ECO57 | |
| P67090|USPD_ECO57_96 | LEIMQKEQCD | 1789,01428 | USPD_ECO57 | |
| P67090|USPD_ECO57_101 | KEQCDLLVCG | 3245,885737 | USPD_ECO57 | |
| P67090|USPD_ECO57_106 | LLVCGHHHSF | 11371,15614 | USPD_ECO57 | |
| P67090|USPD_ECO57_111 | HHHSFINRLM | 34086,79572 | USPD_ECO57 | |
| P67090|USPD_ECO57_116 | INRLMPAYRG | 32652,12137 | USPD_ECO57 | |
| P67090|USPD_ECO57_121 | PAYRGMINKL | 25073,32469 | USPD_ECO57 | |
| P67090|USPD_ECO57_126 | MINKLSADLL | 3788,652408 | USPD_ECO57 | |
| P67090|USPD_ECO57_131 | SADLLIVPFI | 33585,2587 | USPD_ECO57 | |
| Q7ADI5|SUFE_ECO57_1 | MALLPDKEKL | 4922,196593 | SUFE_ECO57 | |
| Q7ADI5|SUFE_ECO57_7 | KEKLLRNFLR | 44580,78197 | SUFE_ECO57 | |
| Q7ADI5|SUFE_ECO57_11 | LRNFLRCANW | 12120,02586 | SUFE_ECO57 | |
| Q7ADI5|SUFE_ECO57_16 | RCANWEEKYL | 2979,836941 | SUFE_ECO57 | |
| Q7ADI5|SUFE_ECO57_22 | EKYLYIIELG | 24299,90705 | SUFE_ECO57 | |
| Q7ADI5|SUFE_ECO57_26 | YIIELGQRLP | 28873,59554 | SUFE_ECO57 | |
| Q7ADI5|SUFE_ECO57_31 | GQRLPELRDE | 5476,536499 | SUFE_ECO57 | |
| Q7ADI5|SUFE_ECO57_36 | ELRDEDRSPQ | 2822,6941 | SUFE_ECO57 | |
| Q7ADI5|SUFE_ECO57_41 | DRSPQNSIQG | 3465,23472 | SUFE_ECO57 | |
| Q7ADI5|SUFE_ECO57_46 | NSIQGCQSQV | 4413,92359 | SUFE_ECO57 | |
| Q7ADI5|SUFE_ECO57_51 | CQSQVWIVMR | 15493,71448 | SUFE_ECO57 | |
| Q7ADI5|SUFE_ECO57_54 | QVWIVMRQNA | 39288,15142 | SUFE_ECO57 | |
| Q7ADI5|SUFE_ECO57_61 | QNAQGIIELQ | 2205,198685 | SUFE_ECO57 | |
| Q7ADI5|SUFE_ECO57_66 | IIELQGDSDA | 1733,815371 | SUFE_ECO57 | |
| Q7ADI5|SUFE_ECO57_71 | GDSDAAIVKG | 4034,312418 | SUFE_ECO57 | |
| Q7ADI5|SUFE_ECO57_76 | AIVKGLIAVV | 72729,73925 | SUFE_ECO57 | |
| Q7ADI5|SUFE_ECO57_81 | LIAVVFILYD | 20721,20037 | SUFE_ECO57 | |
| Q7ADI5|SUFE_ECO57_86 | FILYDQMTPQ | 31851,44333 | SUFE_ECO57 | |
| Q7ADI5|SUFE_ECO57_91 | QMTPQDVVNF | 2707,278199 | SUFE_ECO57 | |
| Q7ADI5|SUFE_ECO57_96 | DVVNFDVRPW | 5215,686617 | SUFE_ECO57 | |
| Q7ADI5|SUFE_ECO57_101 | DVRPWFEKMA | 2322,603736 | SUFE_ECO57 | |
| Q7ADI5|SUFE_ECO57_106 | FEKMALTQHL | 3894,167767 | SUFE_ECO57 | |
| Q7ADI5|SUFE_ECO57_111 | LTQHLTPSRS | 19210,35062 | SUFE_ECO57 | |
| Q7ADI5|SUFE_ECO57_116 | TPSRSQGLEA | 3232,14252 | SUFE_ECO57 | |
| Q7ADI5|SUFE_ECO57_121 | QGLEAMIRAI | 3445,704885 | SUFE_ECO57 | |
| Q7ADI5|SUFE_ECO57_126 | MIRAIRAKAA | 30511,93172 | SUFE_ECO57 | |
|  |  |  |  | |
| Group 3 (separate membrane) | | | | |
| sigma132-141 | QRKLFFNLRK | 32354,88 | RP32_ECO57 | |
| sigma195-205 | MAPVLYLQDKS | 4141,365 | RP32_ECO57 | |
|  |  |  |  | |

**Note**: The spot density of the **sigma_195-205** peptide indicates no or very weak binding to DnaK. This is in contrast to earlier findings [1,2]. A possible reason for this discrepancy could be our required 1h incubation time. This might select for peptides with a slow off rate, high affinity peptides with a fast on and off-rate may not be detected anymore.

Reference List

1. Rodriguez F, Arsene-Ploetze F, Rist W, Rudiger S, Schneider-Mergener J, Mayer MP, Bukau B (2008) Molecular basis for regulation of the heat shock transcription factor sigma32 by the DnaK and DnaJ chaperones. Mol Cell 32: 347-358.

2. Rudiger S, Germeroth L, SchneiderMergener J, Bukau B (1997) Substrate specificity of the DnaK chaperone determined by screening cellulose-bound peptide libraries. Embo Journal 16: 1501-1507.
