# Supplementary material for: Accurate Prediction of DnaK-Peptide Binding via Homology Modelling and Experimental Data
Source: PLoS Comput Biol. 2009 Aug 21;5(8):e1000475. doi: 10.1371/journal.pcbi.1000475 (PMC2717214; doi:10.1371/journal.pcbi.1000475)
Supplement: Table S2 — The various position specific scoring matrices generated and used in the manuscript (0.14 MB DOC) [file pcbi.1000475.s002.doc]

**Table S2a**: Sequence-based PSSM

| Pos/AA | A | C | D | E | F | G | H | I | K | L | M | N | P | Q | R | S | T | V | W | Y |
| --- | --- | --- | --- | --- | --- | --- | --- | --- | --- | --- | --- | --- | --- | --- | --- | --- | --- | --- | --- | --- |
| 1 | -1,58 | -6,14 | -0,37 | -0,08 | -0,04 | -1,24 | 0,21 | 0,11 | 1,00 | -0,17 | -7,97 | 0,63 | -7,64 | 1,28 | -0,73 | 0,37 | 0,55 | -0,33 | 9,06 | 1,46 |
| 2 | -8,41 | -6,36 | -1,02 | -8,66 | 1,11 | -1,14 | -0,15 | 0,74 | 0,86 | 1,21 | -7,64 | 0,12 | -7,64 | -7,46 | 0,48 | -8,01 | -7,75 | 0,16 | -5,44 | 1,87 |
| 3 | -0,65 | -6,14 | -8,28 | -1,60 | 0,55 | -8,12 | -0,15 | -0,15 | -7,89 | 1,55 | -7,64 | -8,01 | -7,64 | -0,62 | 0,62 | -7,89 | -0,29 | 0,82 | 1,46 | 1,82 |
| 4 | -8,41 | -6,14 | -8,22 | -8,49 | -0,33 | -8,41 | -6,83 | 1,50 | -0,79 | 1,53 | 0,60 | -7,84 | -7,69 | -7,39 | -0,33 | -7,93 | 0,26 | -0,30 | -5,44 | -6,70 |
| 5 | -0,76 | -5,44 | -1,24 | -0,98 | 1,06 | -0,76 | -6,70 | -0,36 | -0,89 | 0,57 | 0,29 | -0,73 | 0,29 | -0,62 | 0,84 | -1,10 | -0,04 | 0,72 | -5,44 | 1,57 |
| 6 | -0,09 | -6,36 | -8,25 | -1,58 | 1,57 | -8,41 | -0,15 | 0,21 | 0,55 | 0,45 | 0,29 | 0,55 | 0,55 | 0,65 | -0,48 | -1,06 | 0,62 | -1,53 | 9,76 | 0,95 |
| 7 | -0,12 | -6,36 | -1,17 | -0,55 | 1,46 | -0,01 | 0,45 | -0,58 | -0,37 | -0,24 | -0,24 | 1,24 | -0,55 | -0,48 | 1,40 | -0,48 | 0,84 | -1,53 | -4,75 | 0,95 |

**Table S2b**: Structure-based PSSM

| Pos/AA | A | C | D | E | F | G | H | I | K | L | M | N | P | Q | R | S | T | V | W | Y |
| --- | --- | --- | --- | --- | --- | --- | --- | --- | --- | --- | --- | --- | --- | --- | --- | --- | --- | --- | --- | --- |
| 1 | 0.00 | 0.03 | 0.44 | 1.26 | 0.10 | 0.01 | 0.03 | 0.45 | 0.55 | 0.56 | 0.91 | -0.03 | -0.10 | 0.21 | 0.91 | -0.01 | -0.06 | -0.09 | 1.02 | 0.02 |
| 2 | 0.00 | 0.15 | -0.19 | 0.56 | 1.79 | -0.90 | 0.24 | 2.08 | 1.16 | 1.80 | 2.03 | 0.20 | 0.15 | 0.46 | 1.08 | -0.33 | -0.14 | 0.72 | 3.41 | 1.54 |
| 3 | 0.00 | 0.35 | -0.41 | 1.97 | 3.22 | -0.71 | 0.68 | 0.64 | 0.98 | 2.98 | 3.11 | 0.67 | 0.96 | 1.42 | 1.07 | -0.70 | -0.19 | 0.88 | 1.44 | 3.28 |
| 4 | 0.00 | 0.86 | -1.37 | -1.10 | 0.24 | -0.72 | 0.54 | 3.06 | 0.96 | 4.16 | 4.64 | 0.71 | -0.68 | 1.81 | 1.22 | -0.70 | 1.22 | 2.10 | -4.69 | -5.40 |
| 5 | 0.00 | 0.71 | 0.03 | 0.63 | 1.74 | 0.68 | 0.94 | 1.00 | 1.38 | 1.82 | 2.41 | 0.74 | 1.56 | 1.14 | 1.17 | 0.26 | 0.28 | 0.75 | 3.27 | 1.87 |
| 6 | 0.00 | 0.03 | -0.34 | -0.64 | 1.81 | -0.43 | 0.35 | 1.39 | 0.72 | 1.69 | 1.14 | -0.13 | 0.90 | 0.38 | 0.42 | -0.46 | 0.28 | 1.10 | 0.71 | 1.70 |
| 7 | 0.00 | 0.12 | -0.11 | 0.03 | 0.36 | 0.47 | 0.29 | -0.10 | 0.64 | -0.10 | 0.70 | 0.00 | 1.71 | 0.18 | 0.96 | 0.17 | -0.10 | -0.32 | 0.46 | 0.35 |

**Table S2c**: Final sequence- and structure-based PSSM

| **Pos/AA** | **A** | **C** | **D** | **E** | **F** | **G** | **H** | **I** | **K** | **L** | **M** | **N** | **P** | **Q** | **R** | **S** | **T** | **V** | **W** | **Y** |
| --- | --- | --- | --- | --- | --- | --- | --- | --- | --- | --- | --- | --- | --- | --- | --- | --- | --- | --- | --- | --- |
| **1** | -1,60 | -6,13 | 0,05 | 1,15 | 0,04 | -1,25 | 0,22 | 0,54 | 1,54 | 0,37 | -0,17 | 0,58 | -7,76 | 1,47 | 0,15 | 0,34 | 0,47 | -0,44 | 10,07 | 1,46 |
| **2** | -8,43 | -6,23 | -1,23 | -8,12 | 2,87 | -2,06 | 0,08 | 2,80 | 2,00 | 3,00 | -5,63 | 0,30 | -7,51 | -7,02 | 1,83 | -8,36 | -7,91 | 0,86 | -2,05 | 3,39 |
| **3** | -0,67 | -5,81 | -8,71 | 0,35 | 4,03 | -8,85 | 0,51 | 0,47 | -6,92 | 4,51 | -4,55 | -7,35 | -6,70 | 0,79 | 1,66 | -8,61 | -0,49 | 1,68 | 2,89 | 5,08 |
| **4** | -8,43 | -5,30 | -9,61 | -9,61 | -0,11 | -9,15 | -6,31 | 4,60 | 0,16 | 5,67 | 5,22 | -7,15 | -8,39 | -5,60 | 0,87 | -8,65 | 1,46 | 1,79 | -10,15 | -12,11 |
| **5** | -0,37 | -4,75 | -1,24 | -0,37 | 2,78 | -0,10 | -5,78 | 0,63 | 0,48 | 2,37 | 2,68 | -0,02 | 1,83 | 0,50 | 1,99 | -0,86 | 0,22 | 1,45 | -2,19 | 3,42 |
| **6** | 0,11 | -6,35 | -8,60 | -2,24 | 3,36 | -8,87 | 0,19 | 1,59 | 1,25 | 2,12 | 1,41 | 0,39 | 1,43 | 1,02 | -0,08 | -1,54 | 0,88 | -0,45 | 10,45 | 2,63 |
| **7** | -0,14 | -6,26 | -1,30 | -0,54 | 2,03 | 0,43 | 0,72 | -0,70 | 0,25 | -0,36 | 0,44 | 1,22 | 1,14 | -0,32 | 2,34 | -0,33 | 0,72 | -1,87 | -4,31 | 1,29 |

**Table 2d**: PSSM of Rüdiger *et al* for heptameric peptides (kindly provided by Dr. Bernd Bukau). Note that the original PSSM for 13-mer peptides was reduced here to a 7-mer PSSM. This reduction to score heptapeptides was approved by the original authors of the algorithm.

| Pos/AA | A | C | D | E | F | G | H | I | K | L | M | N | P | Q | R | S | T | V | W | Y |
| --- | --- | --- | --- | --- | --- | --- | --- | --- | --- | --- | --- | --- | --- | --- | --- | --- | --- | --- | --- | --- |
| 1 | 0.11 | -7.31 | -0.66 | -2.22 | -0.21 | 0.5 | 0.36 | -1.56 | 1.28 | -2.55 | -0.12 | -1.11 | -0.23 | 1.7 | 1.79 | 0.2 | 1.37 | 0.39 | 0.65 | -0.29 |
| 2 | -0.79 | -6.35 | -4.91 | -5.14 | 1.17 | -1.95 | -1.74 | 2.05 | -0.4 | 3.62 | -1.1 | -2.36 | -1.63 | -1.6 | 0.79 | -1.27 | -0.27 | 1.75 | -3.49 | 1.88 |
| 3 | -0.79 | -6.35 | -4.91 | -5.14 | 1.17 | -1.95 | -1.74 | 2.05 | -0.4 | 3.62 | -1.1 | -2.36 | -1.63 | -1.6 | 0.79 | -1.27 | -0.27 | 1.75 | -3.49 | 1.88 |
| 4 | -0.79 | -6.35 | -4.91 | -5.14 | 1.17 | -1.95 | -1.74 | 2.05 | -0.4 | 3.62 | -1.1 | -2.36 | -1.63 | -1.6 | 0.79 | -1.27 | -0.27 | 1.75 | -3.49 | 1.88 |
| 5 | -0.79 | -6.35 | -4.91 | -5.14 | 1.17 | -1.95 | -1.74 | 2.05 | -0.4 | 3.62 | -1.1 | -2.36 | -1.63 | -1.6 | 0.79 | -1.27 | -0.27 | 1.75 | -3.49 | 1.88 |
| 6 | -0.79 | -6.35 | -4.91 | -5.14 | 1.17 | -1.95 | -1.74 | 2.05 | -0.4 | 3.62 | -1.1 | -2.36 | -1.63 | -1.6 | 0.79 | -1.27 | -0.27 | 1.75 | -3.49 | 1.88 |
| 7 | -0.69 | -0.38 | -0.53 | -2.48 | -0.8 | -0.05 | -0.14 | -0.17 | 1.62 | 0.03 | -0.26 | 0.44 | 0.42 | 0.23 | 2.58 | 0.35 | 0.72 | -1.05 | -0.18 | -1.73 |
