# Supplementary material for: Accurate Prediction of DnaK-Peptide Binding via Homology Modelling and Experimental Data
Source: PLoS Comput Biol. 2009 Aug 21;5(8):e1000475. doi: 10.1371/journal.pcbi.1000475 (PMC2717214; doi:10.1371/journal.pcbi.1000475)
Supplement: Table S3 — Performance analysis of the predictor on benchmark sets with varying degrees of redundancy (0.04 MB DOC) [file pcbi.1000475.s003.doc]

**Performance analysis of the predictor on benchmark sets with varying degrees of redundancy**

In order to address concerns about the redundancy in the learning and benchmark sets, we have conducted a non-cross-validated and cross-validated performance test with the final matrix on benchmark sets with varying degrees of redundancy from 10% to 100% redundancy, where the 90% redundant set was the set used in the manuscript. Supplementary Table S3 lists for each redundancy threshold the number of sequences left in the negative and positive benchmark sets after redundancy filtering (http://www.expasy.ch/tools/redundancy/).

It can be seen that the largest removal of sequences occurs between 40% and 30% redundancy, indicating that the original benchmark sets are of very low redundancy and are thus well sampled sets. The best Matthews correlation coefficient between 90% and 100% specificity (MCC90) is also given as performance marker in Table S3. Supplementary Figure 1 shows the complete accompanying ROC curves for the non-cross-validated and cross-validated performance tests.

From these results it can be seen that for all benchmark sets of varying redundancy the predictor performs equally well; indicating that the performance estimates are rather independent of the benchmark set composition and redundancy.

**Table S3**: Performance of the best learning set on benchmark sets (BS) with varying degree of redundancy. The table lists for decreasing redundancy thresholds the number of sequences left in the negative and positive benchmark sets after redundancy filtering. The best Matthews correlation coefficient between 90% and 100% prediction specificity (MCC90) is given as performance marker. See also supplementary Figure S1 for accompanying ROC curves.

| BS % redundancy | # seq in negative BS | # seq in positive BS | MCC90 non-crossval | MCC90 crossval |
| --- | --- | --- | --- | --- |
| 100 | 105 | 47 | 0.830 | 0.686 |
| 90 | 100 | 44 | 0.819 | 0.703 |
| 80 | 99 | 42 | 0.812 | 0.668 |
| 70 | 98 | 42 | 0.812 | 0.668 |
| 60 | 98 | 39 | 0.819 | 0.665 |
| 50 | 98 | 39 | 0.819 | 0.665 |
| 40 | 97 | 38 | 0.814 | 0.657 |
| 30 | 56 | 33 | 0.863 | 0.659 |
| 20 | 30 | 22 | 0.847 | 0.658 |
| 10 | 13 | 9 | 0.821 | 0.736 |
