# Supplementary material for: Accurate Prediction of DnaK-Peptide Binding via Homology Modelling and Experimental Data
Source: PLoS Comput Biol. 2009 Aug 21;5(8):e1000475. doi: 10.1371/journal.pcbi.1000475 (PMC2717214; doi:10.1371/journal.pcbi.1000475)
Supplement: Figure S1 — ROC curves to evaluate the performance of the algorithm on benchmark sets with varying degree of redundancy. (0.04 MB DOC) [file pcbi.1000475.s004.doc]

**Figure S1**: ROC curves to evaluate the performance of the final PSSM on benchmark sets with varying degree of redundancy. The legend indicates the % redundancy allowed in the benchmark sets. The curves for 90% and 100% are identical, as well as the curves for 50% and 60% redundancy. Top) Non-cross-validated performance; Bottom) Cross-validated performance.

See also Supplementary Table S3 for benchmark sets sizes and MCC90 values.
